# Proteomics and transcriptomics analyses of Arabidopsis floral buds uncover important functions of ARABIDOPSIS SKP1-LIKE1

**DOI:** 10.1186/s12870-015-0571-9

**Published:** 2016-03-03

**Authors:** Dihong Lu, Weimin Ni, Bruce A. Stanley, Hong Ma

**Affiliations:** Intercollege Graduate Degree Program in Plant Biology, the Huck Institutes of the Life Sciences, the Pennsylvania State University, University Park, PA 16802 USA; Department of Biology, the Pennsylvania State University, University Park, PA 16802 USA; Section of Research Resources, Pennsylvania State University College of Medicine, Hershey, PA 17033 USA; State Key Laboratory of Genetic Engineering and Institute of Plant Biology, Center for Evolutionary Biology, School of Life Sciences, Fudan University, Shanghai, 200433 China; Current address: Department of Plant and Microbial Biology, University of California, Berkeley, CA 94720 USA

**Keywords:** Arabidopsis, ASK1, E3 ubiquitin ligase, Mass spectrometry, Protein degradation, Proteomics, Transcriptomics

## Abstract

**Background:**

The ARABIDOPSIS SKP1-LIKE1 (ASK1) protein functions as a subunit of SKP1-CUL1-F-box (SCF) E3 ubiquitin ligases. Previous genetic studies showed that ASK1 plays important roles in Arabidopsis flower development and male meiosis. However, the molecular impact of ASK1-containing SCF E3 ubiquitin ligases (ASK1-E3s) on the floral proteome and transcriptome is unknown.

**Results:**

Here we identified proteins that are potentially regulated by ASK1-E3s by comparing floral bud proteomes of wild-type and the *ask1* mutant plants. More than 200 proteins were detected in the *ask1* mutant but not in wild-type and >300 were detected at higher levels in the *ask1* mutant than in wild-type, but their RNA levels were not significantly different between wild-type and *ask1* floral buds as shown by transcriptomics analysis, suggesting that they are likely regulated at the protein level by ASK1-E3s. Integrated analyses of floral proteomics and transcriptomics of *ask1* and wild-type uncovered several potential aspects of ASK1-E3 functions, including regulation of transcription regulators, kinases, peptidases, and ribosomal proteins, with implications on possible mechanisms of ASK1-E3 functions in floral development.

**Conclusions:**

Our results suggested that ASK1-E3s play important roles in Arabidopsis protein degradation during flower development. This study opens up new possibilities for further functional studies of these candidate E3 substrates.

**Electronic supplementary material:**

The online version of this article (doi:10.1186/s12870-015-0571-9) contains supplementary material, which is available to authorized users.

## Background

The ubiquitin-proteasome system (UPS) plays important roles in targeted protein degradation, thereby regulating a variety of cellular processes [1–3]. Ubiquitination reactions are catalyzed by the sequential actions of E1 ubiquitin activating enzymes, E2 ubiquitin conjugating enzymes, and E3 ubiquitin ligases. Multiple ubiquitin molecules can be attached to the existing ubiquitin moieties on the protein substrates to form polyubiquitin chains and the polyubiquitinated proteins are usually then degraded by the 26S proteasome.

The UPS regulates many processes in plants, including development and biotic/abiotic stress responses [1, 3–5]. This broad spectrum of functions is made possible by the large number of genes encoding components in the UPS. Plants usually contain a few E1 enzymes, tens of E2 enzymes, and hundreds of E3 ligases, which determine substrate specificities. Therefore, the numerous E3 ligases can potentially ubiquitinate many proteins. Moreover, the modular design of multimeric E3 ubiquitin ligases including the SKP1-CUL1-F-box (SCF) complexes greatly expands the likely number of proteins that can be specifically ubiquitinated. The subunits of SCF complexes are encoded by multi-gene families, especially the F-box proteins, which are encoded by hundreds of genes in plants. Thus, the combination of these components can form various SCF complexes to ubiquitinate numerous substrate proteins.

Genetic studies indicate that plant F-box proteins are involved in hormone signaling pathways, self-incompatibility, developmental processes, and others. Among the F-box proteins important for hormone signaling, TRANSPORT INHIBITOR RESPONSE 1 (TIR1) is a receptor of auxin and the SCF^TIR1^ ubiquitin ligase facilitates the degradation of AUX/IAA proteins, which are repressors of auxin-induced gene expression [6–9]. The F-box protein CORONATINE INSENSITIVE 1 (COI1) has a similar mechanism in regulating jasmonic acid (JA) signaling; COI1 is a receptor of JA and SCF^COI1^ destabilizes JAZ proteins, thereby releasing the transcription factor MYC2 for the activation of JA-responsive genes [10–12]. Other signaling pathways for hormones such as ethylene, gibberellic acid (GA), and abscisic acid (ABA) also require components of the UPS [5]. In addition, S-locus F-box proteins (SLFs) function as the pollen-specific determinants of self-incompatibility [13–16]. The F-box protein UNUSUAL FLORAL ORGANS (UFO) is important for normal meristem identity and floral organ development [17–19]. UFO can interact with LEAFY genetically to activate *AP3* expression [20–22].

The Arabidopsis homolog of the yeast and human *SKP1* genes, the *ARABIDOPSIS SKP1-LIKE1* (*ASK1*), encodes an SCF subunit that bridges Cullin and F-box proteins [23]. It has been shown that ASK1 can interact with F-box proteins UFO [22, 24], COI1 [25], TIR1 [6], and others [24, 26, 27]. Since these F-box proteins have important roles in different pathways, ASK1, as a key component in SCFs, likely has crucial functions in many processes. This was suggested by previous genetic studies of the *ask1* mutant, which has defects in male meiosis, floral organ development, and vegetative growth [23, 28–31]. Although a few substrates of SCFs have been identified in Arabidopsis, they are mainly specific to the well-studied F-box proteins described above. A large number of other ASK1-interacting F-box proteins and their substrates remain elusive, as do the biological pathways regulated by E3s containing ASK1.

Most of the known ubiquitin ligase substrates were identified by protein-protein interaction methods, usually when the F-box protein has a known function [10, 32–34]. Recently, mass spectrometry (MS)-based proteomics approaches have been increasingly applied in various areas including differential gene expression, post-translational modifications, disease marker discovery, as well as the identification of ubiquitin ligase substrates either by detection of ubiquitinated proteins [35, 36], or by comparing proteomes of wild-type (WT) and ubiquitin ligase mutants [37]. In this study, we used a proteomics approach, Multidimensional Protein Identification Technology (MudPIT), to identify floral proteins potentially regulated by ASK1 by comparing floral bud proteomes of WT and *ask1* mutant plants. Furthermore, we performed comparative transcriptomics analysis of WT and *ask1* floral buds to investigate the effect of ASK1 on gene expression. The integrated transcriptomics and proteomics analyses revealed that many proteins are potentially regulated by ASK1-E3s. We discuss several possible ways of how ASK1 might regulate protein stability and further downstream gene expression.

## Results and discussion

### Transcriptomic analysis of L*er* and *ask1* floral buds

**Fig. 1:**
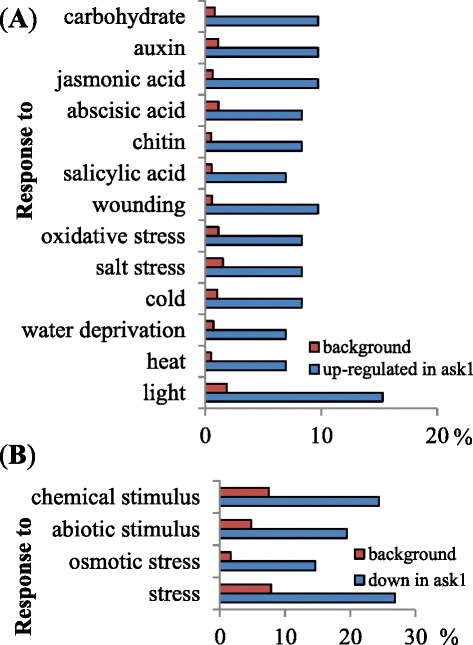
GO categories of stimulus/stress responsive genes enriched in the up-/down-regulated genes in the *ask1* transcriptome. **a** GO categories of stimulus/stress responsive genes enriched in the up-regulated genes in the *ask1* transcriptome. **b** GO categories of stimulus/stress responsive genes enriched in the down-regulated genes in the *ask1* transcriptome. Background percentage (%) represents the proportion of all annotated genes of each GO category within the total genes in the ATH1 microarray. *ask1* percentage (%) represents the proportion of up-/down-regulated genes in the *ask1* transcriptome of each GO category within the total genes in the ATH1 microarray

To determine the effect of the *ask1* mutation on the floral transcriptome, WT (L*er*) and *ask1* floral bud transcriptomes were analyzed using GeneChip Arabidopsis ATH1 Genome Array. The average values from L*er* and *ask1* microarrays were compared to find genes whose RNA levels differ by at least two fold and Student’s t-test p-value < 0.05. We found that 74 and 42 genes were up-regulated and down-regulated, respectively, in *ask1* transcriptome compared with L*er* (Additional files 1 and 2). We used agriGO [38] to determine if certain gene categories are over-represented in the up-/down-regulated genes in *ask1*. We found that genes are enriched in the GO categories of responsive to various stimuli or stresses (Fig. 1). Among the 42 down-regulated genes (including *ASK1*) in *ask1*, 19 genes are related to biotic/abiotic signaling pathways (Table 1), including hormone, light/circadian, temperature, salt, and other signaling pathways. Among the 74 up-regulated genes in *ask1*, 39 genes were annotated to be involved in response to various biotic/ abiotic signals (Table 2). The molecular functions of most of these genes are not well understood except for evidence from transcriptional responses to stimuli (e.g., *COLD*-*REGULATED 15A/15B, DARK INDUCIBLE 10, SENESCENCE 1*, etc.) and sequence homology with well-characterized proteins or protein domains (e.g., HAD superfamily acid phosphatase, *JUMONJI DOMAIN CONTAINING 5*, *CONSTANS-LIKE 2*, etc.).

**Table 1:** Responsive genes down-regulated in the *ask1* mutant transcriptome

Gene ID	Gene name	Signaling pathways/responses
AT5G15960	*KIN1*	Cold and ABA
AT1G35720	*ANNEXIN 1* (*ANNAT1*)	Oxidative stress
AT2G42530	*COLD REGULATED 15B* (*COR15B*)	Cold
AT5G42900	*COLD REGULATED GENE 27* (*COR27*)	Cold
AT2G42540	*COLD*-*REGULATED 15A* (*COR15A*)	Cold
AT4G30650	Low temperature and salt responsive protein	Low temperature and salt
AT5G20250	*DARK INDUCIBLE 10* (*DIN10*)	Light, sucrose
AT1G56220	Dormancy/auxin associated	Dormancy/auxin
AT2G33830	Dormancy/auxin associated	Dormancy/auxin
AT1G28330	*DORMANCY*-*ASSOCIATED PROTEIN*-*LIKE 1*	Dormancy
AT3G20810	*JUMONJI DOMAIN CONTAINING 5* (*JMJD5*)	Circadian
AT5G37260	*CIRCADIAN 1* (*CIR1*)	Circadian
AT4G35770	*SENESCENCE 1* (*SEN1*)	Phosphate starvation
AT3G17790	*PURPLE ACID PHOSPHATASE 17* (*PAP17*)	Phosphate starvation, and hydrogen peroxide
AT1G77120	*ALCOHOL DEHYDROGENASE 1* (*ADH1*)	Anaerobic response
AT2G39920	HAD superfamily acid phosphatase	Cadmium ion
AT4G33020	*ZINC IRON PERMEASE* (*ZIP9*)	Zinc ion
AT5G06870	*POLYGALACTURONASE INHIBITING PROTEIN 2* (*PGIP2*)	Fungal infection, Methyl jasmonate
AT2G05520	*GLYCINE*-*RICH PROTEIN 3* (*GRP3*)	ABA, salicylic acid, ethylene, desiccation

**Table 2:** Responsive genes up-regulated in the *ask1* mutant transcriptome

Gene ID	Gene name/description	Signaling pathways/responses
AT5G54490	*PINOID-BINDING PROTEIN 1* (*PBP1*)	Auxin
AT3G09870	SAUR-like auxin-responsive protein	Auxin
AT5G61600	*ETHYLENE RESPONSE FACTOR 104* (*ERF104*)	Ethylene
AT4G34410	*REDOX RESPONSIVE TRANSCRIPTION FACTOR 1*	Ethylene
AT1G19180	*JASMONATE-ZIM-DOMAIN PROTEIN 1* (*JAZ1*)	Jasmonic acid
AT1G17380	*JASMONATE-ZIM-DOMAIN PROTEIN 5* (*JAZ5*)	Jasmonic acid
AT3G11480	SABATH methyltransferase	Jasmonic acid, fungus, wounding
AT4G27280	Calcium-binding EF-hand family protein	Karrikin
AT3G02380	*CONSTANS-LIKE 2* (*COL2*)	Light
AT3G22840	*EARLY LIGHT-INDUCABLE PROTEIN1* (*ELIP1*)	Light
AT4G14690	*EARLY LIGHT-INDUCIBLE PROTEIN 2* (*ELIP2*)	Light
AT3G17609	*HY5-HOMOLOG* (*HYH*)	Light
AT3G59060	*PHYTOCHROME INTERACTING FACTOR 3-LIKE 6*	Light
AT5G59820	*RESPONSIVE TO HIGH LIGHT 41* (*RHL41*)	Light
AT2G30520	*ROOT PHOTOTROPISM 2* (*RPT2*)	Light
AT2G46830	*CIRCADIAN CLOCK ASSOCIATED 1* (*CCA1*)	Circadian
AT1G01060	*LATE ELONGATED HYPOCOTYL* (*LHY*)	Circadian
AT3G09600	*REVEILLE 8* (*RVE8*)	Circadian
AT3G12580	*HEAT SHOCK PROTEIN 70* (*HSP70*)	Heat
AT5G51440	*HSP20*-like	Heat
AT2G31380	*SALT TOLERANCE HOMOLOGUE* (*STH*)	Salt
AT1G27730	*SALT TOLERANCE ZINC FINGER* (*STZ*)	Salt
AT3G55980	*SALT-INDUCIBLE ZINC FINGER 1* (*SZF1*)	Salt
AT2G33380	*RESPONSIVE TO DESICCATION 20* (*RD20*)	Desiccation
AT5G24660	*RESPONSE TO LOW SULFUR 2* (*LSU2*)	Sulfur deficiency
AT1G19640	*JASMONIC ACID CARBOXYL METHYLTRANSFERASE*	Wounding, and methyljasmonate
AT5G64510	*TUNICAMYCIN INDUCED 1* (*TIN1*)	ER-stress, heat, light, hydrogen peroxide
AT5G57560	*TOUCH 4* (*TCH4*)	Mechanical stimulus
AT1G12110	*NITRATE TRANSPORTER 1.1* (*NRT1.1*)	Nitrate, water deprivation
AT1G61800	*GLUCOSE-6-PHOSPHATE/PHOSPHATE TRANSLOCATOR 2* (*GPT2*)	Glucose, sucrose, karrikin, nematode
AT2G46400	*WRKY DNA-BINDING PROTEIN 46*	Chitin
AT5G51190	Ethylene response factor	Chitin, wounding
AT3G61190	*BON ASSOCIATION PROTEIN 1* (*BAP1*)	Chitin, cold, fungus, heat, jasmonic acid, salicylic acid, wounding
AT4G11280	*ACC SYNTHASE 6* (*ACS6*)	ABA, auxin, chitin, ethylene, jasmonic acid, oxidative stress, wounding
AT5G59310	*LIPID TRANSFER PROTEIN 4* (*LTP4*)	ABA, cold, salt, water deprivation
AT4G25100	*FE SUPEROXIDE DISMUTASE 1* (*FSD1*)	Cadmium ion, copper ion, oxidative stress
AT1G02930	*GLUTATHIONE S-TRANSFERASE 6*	Cadmium ion, oxidative stress, salt, water deprivation
AT3G21890	B-box type zinc finger protein	UV-B, sucrose
AT2G37040	*PHE AMMONIA LYASE 1* (*PAL1*)	UV-B, karrikin, oxidative stress, wounding

Nevertheless, several genes have been functionally characterized, including *CIRCADIAN CLOCK ASSOCIATED 1* (*CCA1*), *LATE ELONGATED HYPOCOTYL* (*LHY*), *JASMONATE*-*ZIM*-*DOMAIN PROTEIN 1* (*JAZ1*), and *JAZ5. CCA1* and *LHY* encode Myb-like transcription factors that synergistically regulate circadian rhythm of Arabidopsis [39] and thus are important for coordinating internal physiological activities with external environmental cues. *JAZ* genes are induced by JA through a feedback loop involving JAZ proteins and the G-box-binding MYC2: JAZ proteins bind to and repress the activity of MYC2 in the absence of JA; upon perception of JA, JAZ proteins are degraded after ubiquitination by SCF^COI1^ and the released MYC2 can activate transcription of downstream genes, including *JAZ* genes [40]. According to this feedback regulatory model, it is expected that the *ask1* mutation would reduce SCF activities, allowing JAZ proteins to accumulate and repress MYC2 activity and thus reducing the JAZ transcript levels. However, we found that *JAZ1* and *JAZ5* transcript levels were unexpectedly higher in the *ask1* mutant than in WT. This paradox suggests that an uncharacterized mechanism may be involved in modulating the JA signaling pathway. For example, ASK1-containing SCFs might facilitate the removal of a yet unidentified transcription activator that has the ability of inducing the expression of *JAZ* genes in the absence of JA; when *ASK1* is mutated this transcriptional activator is stabilized, thereby inducing the expression of downstream genes including *JAZ1* and *JAZ5*. Further studies are needed to uncover new aspects of these regulatory networks.

We then analyzed possible overrepresentation of cis-elements in the putative promoter regions of these up-/down-regulated genes in the *ask1* transcriptome. The frequencies of 6-mer motifs within the 500 bp and 1000 bp putative promoter regions were determined using the Motif Analysis tool from The Arabidopsis Information Resource (TAIR) (Table 3). The G-box (CACGTG) is overrepresented in the putative promoter regions of up-/down-regulated genes, suggesting that corresponding genes might be regulated by G-box-binding transcription factors, which themselves or whose co-factors might be regulated by ASK1-E3 ligases, similar to the JAZ-MYC2 model. Some of these transcription factors or co-factors might be short-lived repressors; when they are stabilized in the absence of ASK1, their target genes are then down-regulated. Others may function as unstable activators, whose stabilization in the absence of ASK1 results in up-regulation of downstream genes. Alternatively, some transcription factors may have dual functions, both activation and repression, as is true for MYC2 [41, 42]. The fact that the genes whose promoters contain these cis-elements are altered in transcription in the *ask1* mutant suggests that the protein levels of the corresponding transcription factors were changed in *ask1*. Another motif, GATAAG (I box), was enriched in the down-regulated genes in *ask1*. The I box was previously found to be enriched in promoters of light-regulated genes [43] and is required for *Arabidopsis rbcS-1A* expression [44]. Further experiments are required to test whether the putative cis-elements are functional and to identify cognate transcription factors that connect ASK1-E3 regulation with transcriptional changes.

**Table 3:** Top five enriched *cis*-elements in the putative promoter regions of down-/up-regulated genes in the *ask1* transcriptome

Oligomer	# in query	# in genomic set	# of promoters in query with oligomer	# of promoters in genomic set with oligoMer	Binomial distribution *p*-value
500 bp promoters of down-regulated genes in *ask1*					
CACGTG	30	7766	12/42	3253/33602	3.53E-04
ACACGT	23	7390	17/42	5609/33602	1.61E-04
CGCAAA	13	4569	13/42	3995/33602	6.16E-04
GCCACG	11	2914	10/42	2594/33602	8.46E-04
GATAAG	27	9179	19/42	7797/33602	9.09E-04
1000 bp promoters of down-regulated genes in *ask1*					
CACGTG	42	12404	16/42	5033/33602	1.57E-04
AACTGT	33	17175	28/42	13171/33602	2.04E-04
GATAAG	41	18464	28/42	13811/33602	4.93E-04
ATTATG	60	33774	35/42	20241/33602	8.37E-04
CGTGTA	22	7824	17/42	6717/33602	1.25E-03
500 bp promoters of up-regulated genes in *ask1*					
CACGTG	72	7766	24/74	3253/33602	4.95E-08
ACACGT	57	7390	30/74	5609/33602	7.19E-07
ACGTGG	30	5475	22/74	4404/33602	9.42E-05
AAGTGG	31	7504	27/74	6502/33602	2.63E-04
ACACTC	23	5788	23/74	5177/33602	3.37E-04
1000 bp promoters of up-regulated genes in *ask1*					
CACGTG	100	12404	30/74	5033/33602	6.83E-08
ACACGT	77	12599	38/74	9080/33602	5.14E-06
ACGTGG	46	9196	31/74	7047/33602	2.50E-05
ATATTA	177	65927	68/74	25116/33602	1.22E-04
TGAGAC	48	12304	36/74	10027/33602	2.98E-04

The enrichment of biotic/abiotic stress related genes in the up-/down-regulated genes in the transcriptome of *ask1* mutant floral buds has several possible implications. First, the up-regulation of 39 biotic/abiotic stress related genes in *ask1* floral buds (Table 2) suggests the expression of such genes might be tightly constrained to avoid unnecessary expression to ensure continuous and maximal allocation of resources to reproductive organs. In WT floral buds, the expression of these genes may be turned off due to degradation of positive transcriptional regulators by ASK1-E3-mediated ubiquitination, but stresses might block the degradation of such positive regulators. Second, the observation that 19 genes annotated as stress responsive were down-regulated in *ask1* floral buds (Table 1) compared with WT floral buds suggests their involvement in normal flower development. Although these genes are annotated as responsive to biotic/abiotic signals, they could be triggered by endogenous signals such as programmed cell death (e.g., tapetum degeneration) and/or controlled dehydration during later stages of anther and pollen development [45]. However, the lack of cell-type-specific transcriptome information makes it difficult to determine the extent to which the transcriptome reprogramming for these developmentally-controlled processes resembles stress responses. In summary, ASK1-E3s might destabilize proteins that are involved in the complex regulations of signaling pathways in normal flower development or in response to external stimuli.

### Proteomic analysis of L*er* and *ask1* floral buds

**Fig. 2:**
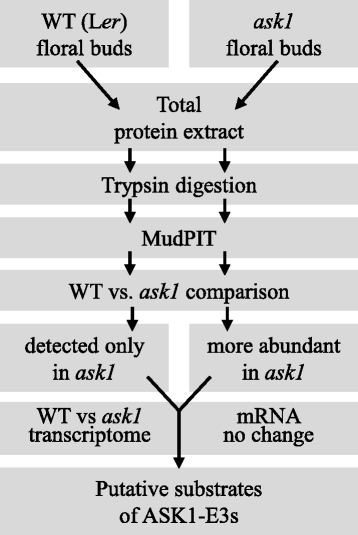
Workflow of the integrated proteomics and transcriptomics to identify putative ASK1-E3 substrates

To probe the effect of *ask1* on the floral proteome and to identify potential substrates of ASK1-E3s, we used a label-free proteomic method, MudPIT, to analyze floral bud proteomes of the *ask1* mutant and L*er* (Fig. 2). Total protein extracts of four L*er* and five *ask1* floral bud samples were digested in-solution with trypsin without pre-separation to maximize digestion of proteins with different properties (e.g., hydrophobicity and charges) and compartmentalization (cytosol, membrane, nucleus and organelles). MudPIT runs of the four L*er* samples (L*er*-1 ~ L*er*_4) detected 2348, 2258, 1658, and 1400 proteins, respectively, with a false discovery rate (FDR) of <1 %, (Additional file 3). When the four datasets were merged, a total of 3220 non-redundant proteins were detected. MudPIT runs of the five *ask1* samples (*ask1*_1 ~ *ask1*_5) detected 1780, 1441, 1959, 1007, and 363 proteins, respectively, with FDR < 1 %, (Additional file 3), for a total of 2916 non-redundant proteins. The *ask1*_5 run detected fewer proteins because the starting protein amount was about 20 % of the others to test whether a smaller amount of input protein extract could lead to different efficiency of protein detection. The test result did not show a huge difference in the detection efficiency when the amount of starting material was changed, i.e., the number of detected proteins is proportional to the starting protein amount. The 363 proteins detected in this test run were included in the total *ask1* proteins, but excluded for comparison between individual runs with spectral counting normalization in the following sections.

**Fig. 3:**
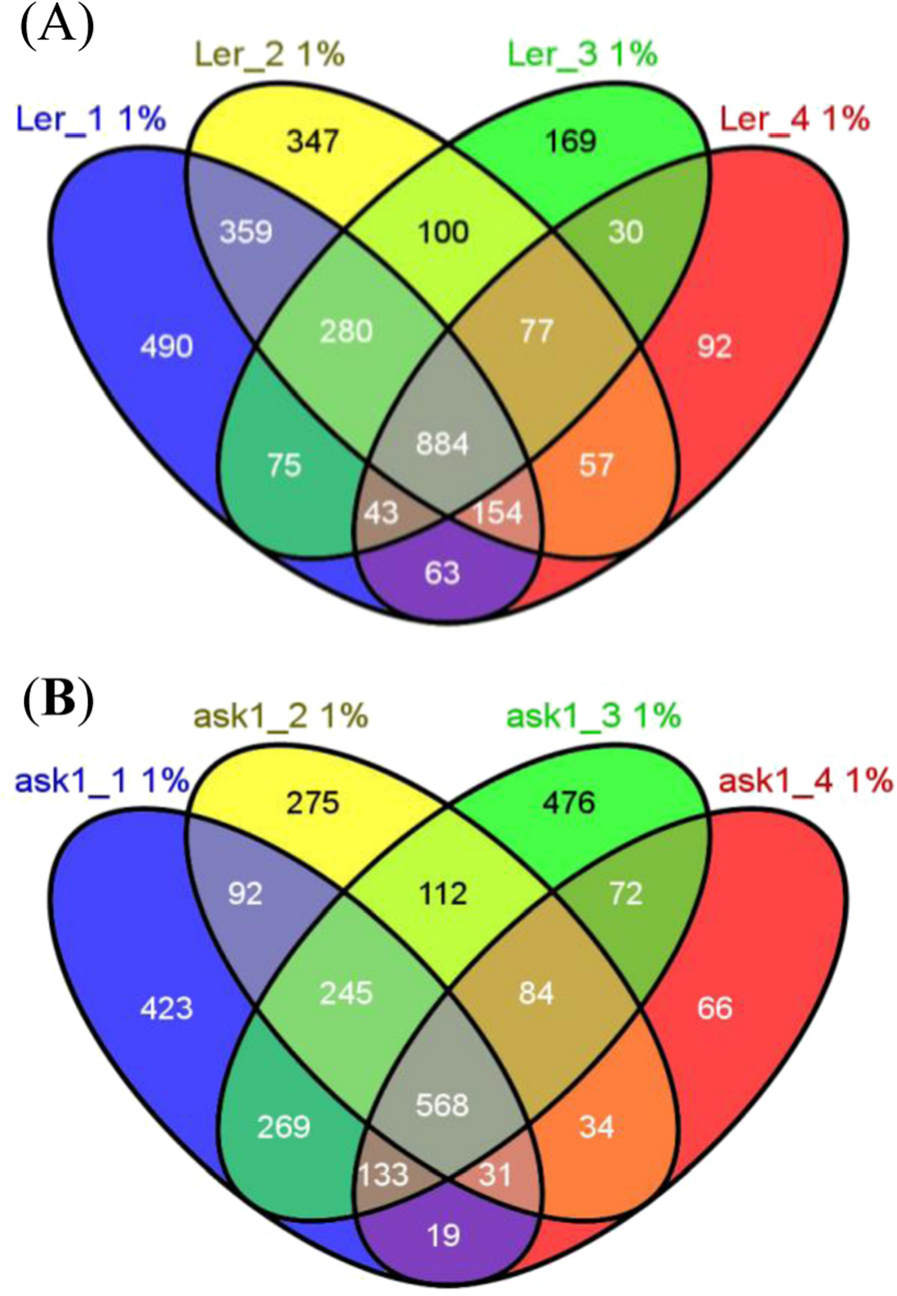
Independent proteomics samples detect partially overlapping sets of proteins. **a** Four L*er* samples L*er*_1 to L*er*_4. **b** Four *ask1* samples *ask1*_1 to *ask1*_4. The “1 %” after each sample name represents FDR < 1 %

The stochastic sampling and detection sensitivity of MS-based proteomics results in the partial identification of the whole proteome in each experiment and partially overlapping datasets from different MS runs. Indeed, analysis of our L*er* and *ask1* samples produced partially overlapping sets of proteins (Fig. 3): 884 (27.5 %) of the 3220 total L*er* proteins (FDR < 1 %) were detected by all four MS runs, 684 (17.2 %) proteins by three MS runs, 554 (21.2 %) proteins by two MS runs, and 1096 (34.1 %) proteins detected only once. Among the 2899 *ask1* proteins (proteins unique to *ask1*_5 not included), 568 (19.6 %), 493 (17.0 %), 598 (20.6 %), and 1240 (42.8 %) proteins were detected by four, three, two and one of the four MS runs, respectively. Even the proteins detected once had high confidence (FDR < 1 %) resulting from very stringent MS detection and searching criteria and thus were regarded as detected.

Proteins of some cellular component categories (e.g., membrane proteins) that are usually considered to be difficult to be detected by MS without using detergents were well represented in our L*er* and *ask1* proteomes (Additional file 4), indicating that our method was able to detect proteins localized to the plasma membrane, organelle membrane, and nuclear envelope. One important improvement to minimize bias was that total protein extracts were digested by trypsin without separation into soluble or insoluble portions. Although insoluble proteins were often thought to be recalcitrant to enzyme digestion, extensive sonication, denaturing treatments, and prolonged tryptic digestion seem to have improved detection efficiency.

### Detection of additional floral proteins compared with previous proteomics studies

**Fig. 4:**
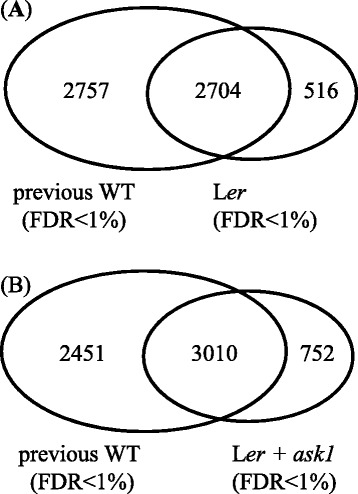
Detection of additional floral bud proteins than previous studies. **a** Comparison of WT floral bud proteins detected in previous studies (previous WT) and L*er* from this study (FDR < 1 %). **b** Comparison of floral bud proteins detected in previous WT and total proteins detected in our L*er* and *ask1* samples (FDR < 1 %)

Comparing our floral bud proteomes with previously published proteomics data, we detected many additional proteins (Fig. 4). WT floral bud proteins from two previous studies [46, 47] were combined into one dataset (named “previous WT”) containing 5461 non-redundant proteins (FDR < 1 %). Compared with the previous WT, additional 516 proteins were only detected in our L*er* proteome dataset containing 3220 non-redundant proteins and additional 752 proteins were detected in our L*er* + *ask1* combined proteome dataset containing 3762 non-redundant proteins. The identification of these additional proteins indicates that proteomics detection has not been saturated. The development of more advanced MS technologies with the capacity of overcoming the huge dynamic range of proteins in biological samples is required to further push the proteomic identification closer to saturation. Until then, we have to keep in mind that there is still much room for improvement to our proteomics data and we need to be cautious when drawing conclusions from the current data.

### Proteins detected only in *ask1* or higher in *ask1*

**Fig. 5:**
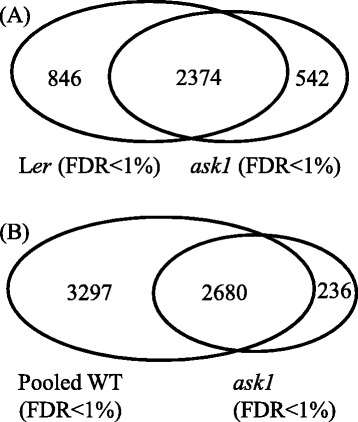
Proteins only detected in *ask1* but not in WT proteomes. **a** Comparison of L*er* and *ask1* proteomes from this study. **b** Comparison of the *ask1* proteome with the pooled WT, a combined WT proteome from previous studies and this study

Comparison of our *ask1* and L*er* proteomes revealed that 542 proteins were found only in *ask1* (Fig. 5a). These proteins have accumulated in *ask1* but not WT, suggesting that they might be putative ASK1-E3 substrates. However, it remains possible that the accumulation of these proteins might be an indirect effect of the *ask1* mutation. Since the limited number of MudPIT runs was not enough to saturate the proteome, we cannot rule out the possibility that some proteins detected only in *ask1* could have been detected also in L*er* if more MS runs had been done. In order to narrow down putative ASK1-E3 substrates, we combined our L*er* floral bud proteome data with the previous WT data [46, 47] yielding a larger WT floral bud proteome dataset consisting of 5977 proteins (Pooled WT). This Pooled WT dataset represents the most comprehensive floral bud proteome currently available obtained by MS methods. The total contribution of our WT (L*er*) floral bud proteome dataset to this Pooled WT proteome is about 53.9 % (3220/5977). By comparing *ask1* and the Pooled WT proteomes, we found that 236 proteins were only detected in *ask1* (*ask1*-only proteins); these are thus more likely to be ASK1-E3 substrates (Fig. 5b and Additional file 5).

We examined RNA levels of these *ask1*-only proteins from microarray data to determine whether increased transcription contributed to the accumulation of these proteins. The RNA levels of most of these genes were not significantly different between *ask1* and L*er* (do not meet the criteria of two-fold cutoff and Student’s t-test p-value < 0.05), except that 11 genes lacked probes on the ATH microarray chip (Additional file 5). Therefore, at least 225 proteins seem to be controlled by ASK1-E3s at the protein level.

Because we used a label-free proteomics method, protein abundance cannot be directly compared between samples. One of the relative quantification approaches, spectral counting, has been demonstrated to show higher reproducibility, larger dynamic range, and stronger linear correlation with relative protein abundance than sequence coverage, peptide number, and ion chromatographic methods [46, 48–51]. Therefore, proteomics datasets of this study were normalized using the spectral counting method as reported [46] and the average values were compared between L*er* and *ask1* to find 322 proteins with higher abundance (1.5-fold cutoff) in *ask1* (*ask1*-higher; Additional file 6). The previous WT data were not included because they were generated by different proteomics methods and difficult to compare quantitatively with our data. We extracted the RNA expression values from microarray data for these *ask1*-higher proteins to determine whether their elevated protein levels were due to increased transcript levels (Additional file 6). Only the RNA level of AT2G33380 was 2.2-fold higher in *ask1* than L*er*. The RNA levels of other genes were not significantly different between *ask1* and L*er*, except that 12 genes had no probes on the microarray chip, suggesting that at least 309 of *ask1*-higher proteins are probably regulated at the protein level.

### ASK1 regulates abundance of regulatory proteins acting at multiple levels

**Fig. 6:**
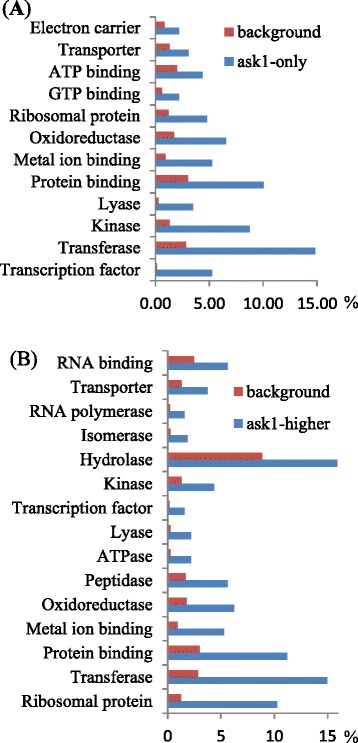
Overrepresented GO categories in proteins accumulated in the *ask1* proteome. **a** Overrepresented GO categories in *ask1*-only proteins. **b** Overrepresented GO categories in *ask1*-higher proteins. Each “Background” percentage (%) represents the proportion of all annotated proteins of each GO category in the total proteins in the *Arabidopsis thaliana* genome (TAIR10 version). Each “*ask1*-only” or “*ask1*-higher” percentage (%) represents the proportion of proteins that were only detected in *ask1* or with higher levels in *ask1* of each GO category in the total proteins detected in the *ask1* samples

GO categorization of the *ask1*-only and *ask1*-higher proteins shows that some molecular functional categories are overrepresented (Fisher test p-value < 0.05) (Fig. 6). Since regulatory proteins are often affected by the UPS, certain categories are of particular interest, including transcriptional regulators, kinases, and peptidases/proteases. Interestingly, many ribosomal proteins were also found to accumulate in *ask1* indicating a possible role of the UPS in translational regulation or extraribosomal functions of ribosomal proteins.

#### ASK1-E3s affects the levels of transcriptional regulators

The *ask1* mutation affected 19 transcription factors and chromatin remodelers, including some with functional information (Table 4). One of them was JAZ9, which might function as a repressor of MYC transcription factors in the JA signaling pathway [52], consistent with the SCF^COI1^-dependent ubiquitination of JAZ proteins and subsequent degradation upon JA perception [10–12]. Another protein detected in *ask1* was the basic helix-loop-helix (bHLH) transcription factor MYC3, which interacts with JAZ proteins and functions with MYC2 and MYC4 to activate JA responses [53, 54]. MYC3 binds to G-boxes [53], possibly regulating promoters with G-boxes, including some of the genes that exhibited altered expression in *ask1,* as supported by the enrichment of G-boxes in the promoters of the genes exhibiting altered expression in *ask1* as described in the first section (Table 3). The accumulation of JAZ9 and MYC3 in the *ask1* proteome raises the possibility that protein stability of both JAZs and MYCs is regulated by the UPS, adding another layer of regulation in the JA signaling pathway. In addition, eight zinc finger transcription factors and several transcription factors of other types (Table 4) also exhibited elevated protein levels in *ask1*, indicating that destabilization of these transcription factors by ASK1-E3s might be important for floral development.

**Table 4:** Transcriptional regulators enriched in *ask1*-only or *ask1*-higher proteins

Transcription factors	
*Enriched in ask1-only proteins*	
AT5G46760	MYC3, basic helix-loop-helix (bHLH) transcription factor
AT1G70700	JASMONATE-ZIM-DOMAIN PROTEIN 9 (JAZ9)
AT1G32360	Zinc finger (CCCH-type) family protein
AT2G24500	Zinc finger (C2H2-type) protein FZF
AT5G60850	Zinc finger OBF BINDING PROTEIN 4 (OBP4)
AT3G61850	Zinc finger DOF AFFECTING GERMINATION 1 (DAG1)
AT4G36620	Zinc finger GATA TRANSCRIPTION FACTOR 19 (GATA19)
AT2G02540	ZINC FINGER HOMEODOMAIN 3 (ZHD3)
AT5G15210	ZINC FINGER HOMEODOMAIN 8 (ZHD8)
AT1G54830	NUCLEAR FACTOR Y, SUBUNIT C3 (NF-YC3)
AT1G58100	TCP DOMAIN PROTEIN 8 (TCP8)
AT3G10490	NAC DOMAIN CONTAINING PROTEIN 52 (ANAC052)
AT4G02020	Polycomb group protein SWINGER (SWN)
*Enriched in ask1-higher proteins*	
AT1G49480	RELATED TO VERNALIZATION1 1 (RTV1)
AT1G76880	Duplicated homeodomain-like superfamily protein
AT3G28920	ZINC FINGER HOMEODOMAIN 9 (ZHD9)
AT3G48430	RELATIVE OF EARLY FLOWERING 6 (REF6); JUMONJI DOMAIN-CONTAINING PROTEIN 12 (JMJ12)
AT4G35570	HIGH MOBILITY GROUP B5 (HMGB5)
AT4G38130	HISTONE DEACETYLASE 1 (HD1);HISTONE DEACETYLASE19

The *ask1* mutation also caused an increased level of a Polycomb group protein, SWINGER (SWN) (Table 4), which interacts with other Polycomb group proteins to repress expression of *FLOWERING LOCUS C* (*FLC*) and controls the initiation of endosperm development [55–57]. The elevated SWN protein level is expected to repress *FLC* expression. Although our *ask1* and L*er* floral bud transcriptome data did not show significant difference of *FLC* expression, our unpublished anther transcriptome data showed that averaged microarray values of *FLC* are 68 and 251 (raw microarray data normalized by R package RMA) in *ask1* and L*er* anther transcriptomes, respectively, i.e., the *FLC* level is lower in *ask1* anthers than that in L*er* anthers (regular Student’s t-test p-value = 0.04). The down-regulation of *FLC* in the *ask1* anther transcriptome suggests that degradation of the SWN protein in developing anthers normally derepress its target genes including *FLC*. Another affected chromatin remodeling protein is RELATIVE OF EARLY FLOWERING 6 (REF6), which is a histone H3 lysine 27 demethylase [58] and positively regulates flowering and brassinosteroid signaling [59]. Our results suggest that ASK1-E3s may modulate gene transcription by facilitating the degradation of both sequence-specific DNA binding transcription factors and chromatin remodelers.

#### ASK1-E3 affects ribosomal proteins

Interestingly, many ribosomal proteins were identified only or with higher levels in *ask1* (Table 5), indicating that ASK1-E3s may also have a role in translational regulation. Several ribosomal proteins have been genetically studied: NUCLEAR FUSION DEFECTIVE 3 is required for polar nuclei fusion during female gametophyte development [60]; PIGGYBACK1 influences leaf vascular patterning [61]; OLIGOCELLULA7 is involved in ribosome biogenesis and organ size control [62]; and POINTED FIRST LEAF2 plays a role in early leaf development [63]. Ribosomal proteins might be regulated by ASK1-E3s either for ribosome turnover, or for extraribosomal regulatory purposes. It was suggested that ribosomal proteins can be ubiquitinated for selective degradation of ribosomes by autophagy [64, 65]. So, the accumulation of ribosomal proteins in *ask1* might result from the failure of ubiquitination by ASK1-E3s and subsequent autophagy. A previous study showed that F-BOX PROTEIN 7 (FBP7), which interacts with ASK1, is required for efficient translation under temperature stress conditions, but the substrate of this F-box protein is not identified [66]. It is possible that F-box proteins, such as FBP7, may regulate translation by ubiquitination of specific ribosomal proteins. Our results suggest that ASK1-E3s might be extensively involved in the regulation of translation.

**Table 5:** Ribosomal proteins enriched in *ask1*-only and *ask1*-higher proteins

Ribosomal proteins	
*Enriched in ask1-only proteins*	
AT5G02610	Ribosomal L29
AT1G26880	Ribosomal protein L34e
AT4G25890	60S acidic ribosomal protein family
AT5G67510	Translation protein SH3-like family protein, large ribosomal subunit
AT5G39850	Ribosomal protein S4
AT5G43640	Ribosomal protein S19
AT4G34555	Ribosomal protein S25
AT3G61110	Ribosomal protein S27
AT1G31817	Mitochondrial 50S ribosomal L21, NUCLEAR FUSION DEFECTIVE 3
AT2G38140	Plastid-specific ribosomal protein 4 (PSRP4)
*Enriched in ask1-only proteins*	
AT1G07830	Ribosomal protein L29 family protein
AT1G15930	Ribosomal protein L7Ae/L30e/S12e/Gadd45 family protein
AT1G26910	Ribosomal protein L16p/L10e family protein
AT1G27400	Ribosomal protein L22p/L17e family protein
AT1G41880	Ribosomal protein L35Ae family protein
AT1G67430	Ribosomal protein L22p/L17e family protein
AT1G69620	RIBOSOMAL PROTEIN L34 (RPL34)
AT1G78630	Ribosomal protein L13 family protein, EMBRYO DEFECTIVE 1473 (EMB1473)
AT2G27530	Ribosomal protein L10aP, PIGGYBACK1 (PGY1)
AT3G07110	Ribosomal protein L13 family protein
AT3G54210	Ribosomal protein L17 family protein
AT3G59540	Ribosomal L38e protein family
AT5G23900	Ribosomal protein L13e family protein
AT5G27850	Ribosomal protein L18e/L15 superfamily protein
AT5G39740	RIBOSOMAL PROTEIN L5B (RPL5B); OLIGOCELLULA 7 (OLI7)
AT2G28830	PLANT U-BOX 12 (PUB12) with ribosomal protein L10e/L16 domain
AT1G74970	RIBOSOMAL PROTEIN S9 (RPS9)
AT2G40510	Ribosomal protein S26e family protein
AT2G40590	Ribosomal protein S26e family protein
AT3G04920	Ribosomal protein S24e family protein
AT3G13120	Ribosomal protein S10p/S20e family protein
AT3G56340	Ribosomal protein S26e family protein
AT4G00100	RIBOSOMAL PROTEIN S13A (RPS13A); POINTED FIRST LEAF 2 (PFL2)
AT4G33865	Ribosomal protein S14p/S29e family protein
AT4G39200	Ribosomal protein S14p/S29e family protein
AT5G04800	Ribosomal S17 family protein
AT5G15200	Ribosomal protein S4
AT5G28060	Ribosomal protein S24e family protein
AT5G52650	RNA binding Plectin/S10 domain-containing protein
AT3G16080	Zinc-binding ribosomal protein family protein
ATCG00800	Chloroplast ribosomal protein S3, RESISTANCE TO PSEUDOMONAS SYRINGAE 3
ATCG01240	30S chloroplast ribosomal protein S7, RIBOSOMAL PROTEIN S7 (RPS7.2)
AT1G07320	Plastid RIBOSOMAL PROTEIN L4 (RPL4); EMBRYO DEFECTIVE 2784 (EMB2784)

Alternatively, ribosomal proteins may have extraribosomal functions, which are exemplified by the previous findings that several ribosomal proteins can block the ubiquitination of the tumor suppressor p53 upon ribosomal stress [67]. Therefore, the ribosomal proteins accumulated in *ask1* might function as regulatory proteins which themselves may be regulated by ubiquitin-mediated proteolysis. Further studies of genetic and molecular interactions between ribosomal proteins and E3 ubiquitin ligases are required for elucidating the role of UPS in translational regulation.

It is difficult to rule out the possibility that the accumulation of these ribosomal proteins might be a side effect of the *ask1* mutation. Since the *ask1* is a stable mutant, it is possible that long-term and large-scale disturbance of protein degradation might impose an intracellular stress which in turn affects the ribosome biosynthesis, activity or turnover. Therefore, more research is needed to elucidate the link between protein degradation and ribosomal protein functions.

#### ASK1-E3s affects regulators of protein activities

A number of kinases accumulated in the *ask1* proteome (Table 6), including some that are important for plant responses to various stimuli. Among them, CALCIUM-DEPENDENT PROTEIN KINASE 6 (CPK6) is a positive regulator of salt/drought stress tolerance [68], methyl jasmonate signaling in guard cells [69], and ABA regulation of guard cell ion channels [70]. In addition, LYSM DOMAIN RECEPTOR-LIKE KINASE 1 (LYSM RLK1) is involved in chitin-mediated plant innate immunity [71, 72], and MAP KINASE KINASE 2 (MKK2) regulates cold and salt stress signaling and innate immunity [73–75]. Our results indicate that ASK1-E3s normally destabilize these kinases during normal flower development, possibly to suppress biotic/abiotic stress responses in the absence of stimuli.

**Table 6:** Kinases enriched in *ask1*-only and *ask1*-higher proteins

Kinases	
*Enriched in ask1-only proteins*	
AT2G17290	CALCIUM-DEPENDENT PROTEIN KINASE 6 (CPK6)
AT4G21940	CALCIUM-DEPENDENT PROTEIN KINASE 15 (CPK15)
AT5G45190	Cyclin T partner CYCT1;5
AT3G48750	Cyclin-dependent kinase CELL DIVISION CONTROL 2 (CDC2)
AT4G29810	MAP KINASE KINASE 2 (MKK2)
AT3G29160	SNF1-RELATED PROTEIN KINASE 1.2 (SnRK1.2)
AT5G63650	SNF1-RELATED PROTEIN KINASE 2.5 (SNRK2.5)
AT4G26100	CASEIN KINASE 1 (CK1)
AT4G35780	ACT-like protein tyrosine kinase
AT5G49470	PAS domain-containing protein tyrosine kinase
AT5G11020	Protein kinase superfamily protein
AT5G24010	Protein kinase superfamily protein
AT5G57610	Protein kinase superfamily protein
AT5G43020	Leucine-rich repeat protein kinase family protein
AT3G21630	LYSM DOMAIN RECEPTOR-LIKE KINASE 1 (LYSM RLK1)
AT3G14350	STRUBBELIG-RECEPTOR FAMILY 7 (SRF7)
AT4G33240	1-phosphatidylinositol-3-phosphate (PtdIns3P) 5-kinase
*Enriched in ask1-higher proteins*	
AT1G31910	GHMP kinase family protein
AT2G18170	MAP KINASE 7 (ATMPK7)
AT2G27970	CDK-SUBUNIT 2 (CKS2)
AT3G02880	Leucine-rich repeat protein kinase family protein
AT4G21210	PPDK REGULATORY PROTEIN (RP1)
AT4G35230	BR-SIGNALING KINASE 1 (BSK1)

Also affected are two cell cycle regulators, cyclin-dependent kinase CELL DIVISION CONTROL 2 (CDC2) and a Cyclin T protein, CYCT1;5. CDC2 is required for male gametogenesis [76]. CYCT1;5 is a subunit of cyclin-dependent kinase C complexes involved in cauliflower mosaic virus infection, plant growth and development [77]. The accumulation of cell cycle regulators in *ask1* may affect mitosis and/or meiosis, as suggested by the findings that *ask1* mutant plants have reduced cell numbers and defective male meiosis [23, 28–31]. Timely removal of cell cycle regulators is likely to be an important part of ASK1 function in regulating plant development.

We also found that SNF1-RELATED PROTEIN KINASE 1.2 (SnRK1.2)/SNF1 KINASE HOMOLOG 11 (KIN11), which was reported to be degraded during phosphate starvation [78], accumulated in the *ask1* proteome. SnRK1.2/KIN11 was also shown to interact with ASK1 [79]. These findings imply that ASK1 might directly recruit SnRK1.2/KIN11 to Cul1 without an F-box protein, resulting in ubiquitination and degradation of SnRK1.2/KIN11. Alternatively, an F-box protein might also interact with ASK1 and SnRK1.2/KIN11, forming an SCF complex for ubiquitinating SnRK1.2/KIN11.

Other kinases are largely unknown, but might have important functions in signal perception and transduction. For example, AT5G43020 and AT3G14350 contain transmembrane domains and could be membrane receptor kinases. In summary, the increased levels of these kinases suggest that ASK1-E3s negatively regulate levels of these protein kinases to control cell cycle, plant immunity, hormone signaling, and other processes.

#### ASK1-E3 affects regulators of protein stability

The peptidase category is enriched in the *ask1*-higher proteins (Table 7), indicating that ASK1-E3s may regulate degradation of peptidases, which in turn affect protein processing or turnover. Four peptidases (AT1G53850, AT5G66140, AT1G77440, and AT3G60820) are isoforms of 20S proteasome alpha/beta subunits, indicating that the proteasome core complex may also be regulated by UPS. Two ubiquitin-specific proteases UBIQUITIN-SPECIFIC PROTEASE5 (UBP5) and UBP6 were also detected in *ask1*-higher proteins, suggesting that deubiquitinases, which antagonize protein ubiquitination, might also be regulated by the UPS. The BRI1 SUPPRESSOR 1 (BRS1), a secreted serine carboxypeptidase, is involved in brassinosteroid signaling possibly by processing some proteins [80]. Other peptidases are largely unknown except information from expression and homology. Peptidases/proteases may normally be subject to negative regulation by ASK1-E3s, thus coupling peptidase-mediated protein processing or degradation with the UPS.

**Table 7:** Peptidases enriched in *ask1*-higher proteins

Peptidases	
AT1G01300	Eukaryotic aspartyl protease family protein
AT1G79720	Eukaryotic aspartyl protease family protein
AT1G02305	Cysteine proteinases superfamily protein
AT3G62940	Cysteine proteinases superfamily protein
AT5G43060	Granulin repeat cysteine protease family protein, ESPONSIVE TO DEHYDRATION 21B (RD21B)
AT4G30610	SERINE CARBOXYPEPTIDASE 24 PRECURSOR (SCPL24); BRI1 SUPPRESSOR 1 (BRS1)
AT4G30810	SERINE CARBOXYPEPTIDASE-LIKE 29 (SCPL29)
AT1G13270	METHIONINE AMINOPEPTIDASE 1B (MAP1C)
AT3G14067	Subtilase family protein
AT5G04710	Zn-dependent exopeptidases superfamily protein
AT5G05740	S2P-like putative metalloprotease, ETHYLENE-DEPENDENT GRAVITROPISM-DEFICIENT AND YELLOW-GREEN-LIKE 2 (EGY2)
AT2G40930	UBIQUITIN-SPECIFIC PROTEASE 5 (UBP5)
AT1G51710	UBIQUITIN-SPECIFIC PROTEASE 6 (UBP6)
AT1G53850	20S PROTEASOME ALPHA SUBUNIT E1 (PAE1)
AT5G66140	20S PROTEASOME ALPHA SUBUNIT D2 (PAD2)
AT1G77440	20S PROTEASOME BETA SUBUNIT C2 (PBC2)
AT3G60820	20S PROTEASOME BETA SUBUNIT F1 (PBF1)

### Possible ways that ASK1 regulates gene expression

**Fig. 7:**
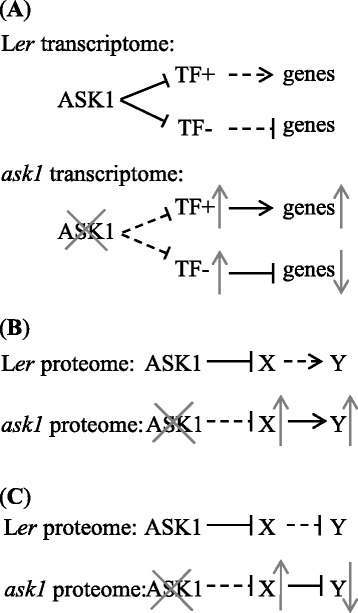
Possible mechanisms of transcriptome and proteome regulations by ASK1-E3s. **a** ASK1-E3s may regulate gene transcription by destabilizing transcription factors. The transcription factors are stabilized in *ask1* mutant and activate or repress downstream gene transcription. TF+, transcriptional activators; TF-, transcriptional repressors. **b** ASK1-E3s might destabilize substrate X, which positively regulates the abundance of target proteins Y. In the *ask1* mutant proteome, ASK1-E3 substrate X and their target protein Y accumulate. **c** ASK1-E3s might destabilize substrate X, which negatively regulates the abundance of target protein Y. In the *ask1* mutant proteome, ASK1-E3 substrate X accumulates but target protein Y decreases. Bars, negative regulation; horizontal arrows, positive regulation; dashed gray bars and horizontal arrows, missing regulations; upward arrows, increase in abundance; downward arrows, decrease in abundance

By integrative analysis of transcriptome and proteome data, we found that ASK1-E3s might regulate gene expression at multiple steps, ranging from transcriptional, translational, to post-translational regulations. ASK1-E3s may destabilize transcription repressors or activators to derepress or inactivate gene transcription, respectively (Fig. 7a). In the absence of ASK1, the accumulation of these transcriptional repressors or activators results in down-regulation or up-regulation of gene transcription, respectively. However, we cannot rule out the possibility that the altered transcriptome and proteome might be indirect consequences of the *ask1* mutation.

The proteins accumulated in *ask1* might be direct substrates of ASK1-E3s, or stabilized by ASK1-E3 substrates (Fig. 7b). For example, ubiquitin-specific proteases UBP5 and UBP6, which accumulate in the *ask1* proteome (Table 7), might be substrates of ASK1-E3s; UBP5 and UBP6 could deubiquitinate and prevent degradation of ubiquitinated proteins, whose protein levels are then increased in *ask1*. An example in human is the herpesvirus-associated ubiquitin-specific protease (HAUSP), which stabilizes a tumor suppressor p53 by deubiquitination [81]. Ribosomal proteins may share a similar mechanism: accumulation of ribosomal proteins in *ask1* may increase protein synthesis; alternatively, if ribosomal proteins have extraribosomal regulatory functions, they may stabilize some proteins in a similar way as those stabilizing p53 in human [67].

In another possible scenario, ASK1-E3s may destabilize some proteolytic enzymes (e.g., E3 ubiquitin ligases or peptidases), which can degrade other proteins (Fig. 7c), forming a double negative regulation cascade. The accumulation of such proteolytic enzymes in *ask1* may cause reduced levels of their proteolytic substrates. Proteasome subunits and peptidases that accumulate in *ask1* may be involved in degradation of their substrate proteins, which could be detected with lower levels in *ask1* proteome (Additional file 7). However, it remains difficult to identify these proteolytic substrates due to lack of functional information of the proteolytic enzymes.

There are probably more proteins regulated by ASK1-E3s than those identified in this study. For example, the substrates of the well-studied F-box proteins, TIR1 and COI1, were not detected except JAZ9 (Table 4). One possible reason is that, due to technical limitations, MS might not have uncovered proteins with low-level and/or spatiotemporally restricted expression (e.g, the putative UFO substrate, LEAFY, which is mainly expressed in the inflorescence meristem and floral meristem [20–22]), and when the substrates of F-box proteins are subject to degradation. Another important reason is the functional redundancies among the 21 *ASK* family members in Arabidopsis. Since the *ASK1* gene is expressed throughout the plant with higher levels in growing organs, its mutation is expected to cause more defects in many plant organs. However, the actual defects are milder than the expected, probably due to the redundancy among the *ASK* family members. The *ASK2* gene is the most closely related gene to *ASK1*. The single mutant of *ask2* is similar to WT plants, but the *ask1 ask2* double mutant has severe defects in embryo development and is lethal soon after seed germination [82]. This suggests that the redundancy of ASK1 with ASK2, and possibly other ASK proteins, probably has masked some aspects of the ASK1 function. In other words, some ASK1-E3 substrates might also be ubiquitinated by SCFs containing other ASK proteins (e.g., ASK2-E3s), and thus would be unable to accumulate in the *ask1* proteome. This might also explain why most of the well-known substrates of F-box proteins (TIR1 and COI1) were not identified in our MS data.

For example, one new aspect of ASK1 functions was revealed by our reanalysis of female fertility of the *ask1* mutant, which was reported to be female fertile in previous studies [23, 28–31]. We loaded excess WT (L*er*) pollen onto stigmas of the *ask1* mutant, the *dysfunctional tapetum 1* (*dyt1*) mutant **(**as a control with male sterility and female fertility) [83], and L*er* (as a self-pollination control), and finally we counted mature seeds from every silique (Additional file 8). The result clearly showed that the pollinated *ask1* pistils yielded significantly fewer seeds (16.0 seeds/silique on average) than L*er* (52.5 seeds/silique on average) and *dyt1* (52.0 seeds/silique on average) (Student’s t-test p-value < 0.01); while the pollinated *dyt1* pistils yielded similar numbers of seeds as L*er* (Student’s t-test p-value > 0.05). This finding suggests a previously unrecognized role of ASK1 in female reproductive development in Arabidopsis. Studying the masked aspects of ASK1 functions will need tissue-specific silencing of multiple *ASK* family members, or tissue-specific *ASK1* complementation within the *ask1 ask2* double mutant or higher order mutants. In addition, characterization of the ubiquitinated proteome may identify potential substrates of E3 ubiquitin ligases and ubiquitination sites within each protein, providing additional clues about ASK1 function in related processes.

## Conclusions

Protein degradation is an integral part of various biological processes. The UPS is of particular interest since it selectively degrades proteins, including many key regulators of many cellular pathways [1–3]. However, searching for specific substrates of E3 ubiquitin ligases has been difficult probably due to rapid degradation of substrate proteins once they have been polyubiquitinated by E3 ubiquitin ligases, relatively weak interaction between E3s and substrates, narrow spatiotemporal window where the E3-substate interaction occurs, and others.

In this study, we have searched for potential E3 substrates by using an Arabidopsis mutant that lacks the functional *ASK1* gene encoding a key component of SCF-type E3 ubiquitin ligases and that has developmental defects, particularly in floral organs including petals and anthers [23, 28–31]. We employed a MS-based method, MudPIT, to explore floral bud proteomes and detected 2916 and 3220 proteins in *ask1* and WT proteomes, respectively. By comparing the *ask1* proteome with a pooled WT floral bud proteome (our WT floral bud proteome combined with two published WT floral bud proteomes), we found 236 proteins that are unique to the *ask1* proteome and 322 proteins with higher levels in the *ask1* proteome. The accumulation of these proteins in the absence of ASK1-E3s suggests that they may be targeted by ASK1-E3s for degradation in WT. Our transcriptomics analysis of *ask1* and WT floral buds showed that the transcripts of genes encoding the proteins accumulated in the *ask1* proteome are not significantly affected by the *ask1* mutation, suggesting that these proteins are regulated at the protein level and thus are more likely to be candidate substrates of ASK1-E3s. Functional categorization revealed that many of the potential substrates of ASK1-E3s are involved in regulation of transcription, translation, protein phosphorylation, and protein degradation. This indicates a multifaceted role of ASK1 in regulating plant development. Much more work is required to validate these candidate E3 substrates and to investigate their specific molecular functions.

## Methods

### Plant materials and growth conditions

The *Arabidopsis thaliana* ecotype Landsberg *erecta* (L*er*) and *ask1* mutant within the L*er* background [23] were used. Plants were grown on soil (Metro-Mix 360, Sun Gro Horticulture, Bellevue, WA) in a growth room with a temperature of 23 °C and long day conditions (16 h light and 8 h dark). The *ask1* mutant plants were selected from the progeny of *ASK1*/*ask1* heterozygous plants by their abnormal phenotypes including reduced plant size compared with WT plants of the same age, reduced number and/or reduced size of petals, sterile anthers, short filaments, and short siliques. Clusters of unopened floral buds from the primary inflorescences (from inflorescence meristem to the biggest unopened bud) of the *ask1* mutant and L*er* were collected from plants with about 5 open flowers.

### Microarray analysis

L*er* and *ask1* floral bud total RNA was extracted using the NucleoSpin® RNA Plant kit (MACHEREY-NAGEL, Bethlehem, PA). RNA quality analysis was performed on the Agilent 2100 Bioanalyzer (Agilent Technologies, Santa Clara, CA), controlled by the Agilent 2100 Expert software, using the Plant RNA Nano assay following the RNA 6000 Nano kit protocol. Microarray was performed using the GeneChip Arabidopsis ATH1 Genome Array (Affymetrix, Santa Clara, CA) in the Penn State Genomics Core Facility – University Park, PA. Three biological replicates of *ask1* and four biological replicates of L*er* were performed (Additional file 9).

Data analysis was conducted as previously described with some modifications [84]. Microarray datasets (.CEL files) were normalized by R package RMA and exported as Excel files. Microarray signal values were averaged from biological replicates of each genotype and compared between *ask1* and L*er* to find differentially expressed genes which show at least 2-fold differences in RNA levels and p-value < 0.05 (regular Student’s t-test). GO categorization was conducted using the Singular Enrichment Analysis (SEA) from agriGO [38]. The Affymetrix ATH1 Genome Array (GPL198) was selected as the background reference which contains 22479 annotated genes. The statistical test was set to Fisher and significance level set to 0.05.

### Protein extraction with trichloroacetic acid/acetone method

The protein extraction method was modified from a previous study [85]. Floral buds were ground thoroughly in liquid nitrogen with mortars and pestles and the powder was suspended in -20 °C Acetone with 10 % w/v Trichloroacetic Acid (TCA) and 0.07 % (v/v) β-Mercaptoethanol (1 ml for 0.3 g of tissue powder). After being incubated for 2 h (or overnight) at -20 °C, the protein suspension was centrifuged for 15-20 min at 14,000 rpm. The supernatant was removed and the protein pellet was resuspended and washed with 1 ml of -20 °C Acetone containing 0.07 % (v/v) β-Mercaptoethanol followed by centrifugation for 15-20 min at 14,000 rpm. This washing step was repeated until the pellet was almost white. The protein pellet was vacuum dried for 5-10 min and stored at -20 °C or immediately used for trypsin digestion.

### In-solution trypsin digestion of protein extract

About 20-30 mg of crude protein extract from the TCA/Acetone method was resuspended in 1 ml of rehydration buffer [100 mM NH_4_HCO_3_, 10 mM Dithiothreitol (DTT), 10 % (v/v) Acetonitrile] and sonicated for 5 times, 20 s each time, duty cycle 40 %, power 3 using a Branson Sonifier S-450A (Branson Ultrasonics, Danbury, CT). Proteins were denatured at 60 °C for 45–60 min and alkylated by 50 mM Iodoacetamide at 37 °C for 30 min in dark. 40 μl of 1 M DTT was added to quench the alkylation reaction. Alkylated proteins were digested by 20 μg of Trypsin Gold, Mass Spectrometry Grade (Promega, Madison, WI) for 16-18 h at 37 °C with moderate shaking. The remaining indigestible debris was removed by centrifugation at 12,000 rpm for 10 min. The supernatant was transferred to a new 1.5 ml tube and centrifuged again to remove residual debris. The supernatant was transferred to a new 1.5 ml tube and was adjust to pH 3.0 with glacial acetic acid. The peptide solution was vacuum dried completely to evaporate off NH_4_HCO_3_ and acetonitrile. The pellet was resuspended in 200 μl of H_2_O and vacuum dried. Three repeats of resuspension and drying were performed in total. Finally the peptides were analyzed in the Proteomics and Mass Spec Core Facility, College of Medicine, Pennsylvania State University, Hershey, PA.

### Mass spectrometry analysis/MudPIT

Trypsin-digested peptide samples were analyzed by MudPIT according to the 2D LC-MALDI separation and analysis procedures published previously using a 4800 proteomic analyzer MALDI TOF/TOF tandem system (Applied Biosysems) [86] except several modifications. The ProteinPilot software version 4.2 was used to perform protein identification by searching MS spectra against the protein database which included the *Arabidopsis thaliana* protein list TAIR10_pep_20101214, 156 common human and lab contaminants (ABSciex_ContaminantDB_20070711), and a reverse “decoy” version of the protein database itself (concatenated Reverse Decoy Database). Proteins with local FDR < 1 % were accepted as detected (Additional files 10, 11, 12, 13, 14, 15, 16, 17, and 18).

### Proteomics data analysis

We combined proteins detected in *ask1* samples into the *ask1* proteome, and combined proteins detected in L*er* into the L*er* proteome. We first compared our *ask1* and L*er* proteomes to find proteins that are only detected in *ask1* samples. We also obtained previously published proteomics data of wild-type *Arabidopsis thaliana* floral buds [46, 47] and combined them into a “previous WT” proteome containing 5461 non-redundant proteins (FDR < 1 %). Comparison of our L*er* and *ask1* proteomes with the previous WT proteome resulted in the finding of additional floral proteins in our data. We combined our L*er* floral bud proteome with the previous WT proteome [46, 47] to a “Pooled WT” proteome consisting of 5977 non-redundant proteins. Comparison of our *ask1* proteome with the Pooled WT proteome led to the identification of proteins that are considered as “*ask1*-only” proteins.

Since each sample was analyzed by MudPIT individually without labeling and multiplexing, the abundance of each protein cannot be directly compared across different samples. Instead, the relative abundance of each protein in a sample was normalized using the spectral counting method as previously described [46, 48–51, 87]. The following formula is used to calculate the spectral counting values which represent the normalized relative abundance of proteins:

$$ \mathrm{Abundance}\kern0.5em \mathrm{of}\kern0.5em \mathrm{protein}\kern0.5em \mathrm{K}=\frac{\left[\frac{\left(\mathrm{Measured}\kern0.5em \mathrm{s}\mathrm{pectra}\kern0.5em \mathrm{of}\kern0.5em \mathrm{protein}\kern0.5em \mathrm{K}\right)}{\mathrm{Measured}\kern0.5em \mathrm{s}\mathrm{pectra}\kern0.5em \mathrm{of}\kern0.5em \mathrm{all}\kern0.5em \mathrm{protein}\mathrm{s}\kern0.5em \mathrm{in}\kern0.5em \mathrm{dataset}}\right]}{\left[\frac{\mathrm{Theoretical}\kern0.5em \mathrm{peptides}\kern0.5em \mathrm{of}\kern0.5em \mathrm{protein}\kern0.5em \mathrm{K}}{\mathrm{Theoretical}\kern0.5em \mathrm{peptides}\kern0.5em \mathrm{of}\kern0.5em \mathrm{all}\kern0.5em \mathrm{protein}\mathrm{s}\kern0.5em \mathrm{in}\kern0.5em \mathrm{dataset}}\right]} $$

The “Measured spectra of protein K” is the number of actually detected MS spectra that specifically match to the protein K.

The “Measured spectra of all proteins in dataset” is the sum of the measured spectra of proteins in one sample.

The “Theoretical peptides of all proteins in dataset” is the total number of the *in silico* tryptic peptides of all proteins detected in one sample. The *in silico* tryptic digestion was carried out using the tool “digest” from the Galaxy platform (https://usegalaxy.org/). Since trypsin normally does not cut after lysine (K) or arginine (R) residues if it is followed by a Proline (P), we specified these sites as non-cut sites. Partial digestion and fragments containing more than one potential cut site were not included. Peptides containing at least 6 amino acid residues were considered as theoretical peptides.

The “Theoretical peptides of protein K” is the number of tryptic peptides of a protein K that was determined in the above “Theoretical peptides of all proteins in dataset”.

For a protein detected in both *ask1* and L*er* samples, its spectral counting values were averaged across *ask1* and L*er* samples, respectively. Then the average spectral counting values of a protein in *ask1* and L*er* samples were compared. Proteins whose average spectral counting value in *ask1* samples is at least 1.5-fold of that in L*er* samples were considered as “*ask1*-higher” proteins.

### Availability of supporting data

The data sets supporting the results of this article are included within the article and its additional file. The raw microarray datasets were deposited in the National Center for Biotechnology Information (NCBI) Gene Expression Omnibus (GEO) with the accession number GSE42841.

## Additional files

Additional file 1: 
**Genes** **up-regulated** **in the** ***ask1*** **mutant microarray compared with L** ***er.***
Additional file 2: 
**Genes** **down-regulated** **in the** ***ask1*** **mutant microarray compared with L** ***er.***
Additional file 3: 
**Proteins detected in individual proteomics samples with FDR < 1 %.**
Additional file 4: 
**Three membrane protein categories are well represented in the detected L** ***er*** **and** ***ask1*** **proteins (FDR < 1 %).**
Additional file 5: 
***ask1*** **-only proteins with microarray values.**
Additional file 6: 
***ask1*** **-higher proteins with spectral counting and microarray values.**
Additional file 7: 
***ask1*** **-lower proteins with spectral counting and microarray values.**
Additional file 8: 
**The** ***ask1*** **mutant has reduced female fertility.**
Additional file 9: 
**Normalized microarray data of three biological replicates of** ***ask1*** **and four biological replicates of L** ***er*** **floral buds.**
Additional file 10: 
**Mass spectrometry data of** ***ask1*** **_1 sample exported as .zip file.**
Additional file 11: 
**Mass spectrometry data of** ***ask1*** **_2 sample exported as .zip file.**
Additional file 12: 
**Mass spectrometry data of** ***ask1*** **_3 sample exported as .zip file.**
Additional file 13: 
**Mass spectrometry data of** ***ask1*** **_4 sample exported as .zip file.**
Additional file 14: 
**Mass spectrometry data of** ***ask1*** **_5 sample exported as .zip file.**
Additional file 15: 
**Mass spectrometry data of L** ***er_*** **1 sample exported as .zip file.**
Additional file 16: 
**Mass spectrometry data of L** ***er_*** **2 sample exported as .zip file.**
Additional file 17: 
**Mass spectrometry data of L** ***er_*** **3 sample exported as .zip file.**
Additional file 18: 
**Mass spectrometry data of L** ***er_*** **4 sample exported as .zip file.**

## Abbreviations

ASK1: ARABIDOPSIS SKP1-LIKE1
SCF: SKP1-CUL1-F-box
ASK1-E3s: ASK1-containing SCF E3 ubiquitin ligases
UPS: Ubiquitin-proteasome system
TIR1: TRANSPORT INHIBITOR RESPONSE 1
COI1: CORONATINE INSENSITIVE 1
JA: Jasmonic acid
GA: Gibberellic acid
ABA: Abscisic acid
SLFs: S-locus F-box proteins
UFO: UNUSUAL FLORAL ORGANS
MS: Mass spectrometry
MudPIT: Multidimensional Protein Identification Technology
CCA1: CIRCADIAN CLOCK ASSOCIATED 1
LHY: LATE ELONGATED HYPOCOTYL
JAZ1: JASMONATE-ZIM-DOMAIN PROTEIN 1
TAIR: The Arabidopsis Information Resource
FDR: False discovery rate
WT: Wild-type
bHLH: Basic helix-loop-helix
SWN: SWINGER
FLC: FLOWERING LOCUS C
REF6: RELATIVE OF EARLY FLOWERING 6
FBP7: F-BOX PROTEIN 7
CPK6: CALCIUM-DEPENDENT PROTEIN KINASE 6
LYSM RLK1: LYSM DOMAIN RECEPTOR-LIKE KINASE 1
MKK2: MAP KINASE KINASE 2
CDC2: Cell Division Cycle 2
SnRK1.2: SNF1-RELATED PROTEIN KINASE 1.2
KIN11: SNF1 KINASE HOMOLOG 11
UBP: UBIQUITIN-SPECIFIC PROTEASE
BRS1: BRI1 SUPPRESSOR 1
HAUSP: Herpesvirus-associated ubiquitin-specific protease
DYT1: DYSFUNCTIONAL TAPETUM 1
L*er*: Landsberg *erecta*
SEA: Singular Enrichment Analysis
NCBI: National Center for Biotechnology Information
GEO: Gene Expression Omnibus
TCA: Trichloroacetic Acid
DTT: Dithiothreitol
